# How effective are digital interventions in increasing flu vaccination amongst pregnant women? A systematic review protocol

**DOI:** 10.1186/s13643-020-01372-z

**Published:** 2020-05-28

**Authors:** Joanne Parsons, Helen Atherton

**Affiliations:** Unit of Academic Primary Care, University of Warwick, Gibbet Hill, Coventry, CV4 7AL UK

**Keywords:** Systematic Review, Flu vaccination, Pregnancy, Digital interventions

## Abstract

**Background:**

Pregnant women and their unborn babies are at increased risk of complications as a result of flu, yet uptake of the flu vaccination in the UK remains low. Digital interventions have proven effectiveness in changing health behaviour, but their effectiveness in increasing flu vaccination amongst pregnant women has not been examined. This protocol details the design and methodology of a systematic review and meta-analysis, examining the effectiveness of digital interventions in increasing flu vaccination amongst pregnant women.

**Methods:**

Bibliographic databases will be searched using appropriate search terms related to vaccination, pregnancy and flu. Randomised, non-randomised, quasi randomised controlled trials and other quantitative study designs will be eligible for inclusion, and studies will present the rate of flu vaccination amongst pregnant women of digital interventions compared to non-digital interventions, or usual care. No date or study country restrictions will be put on included studies, but studies will be published in English.

**Discussion:**

This is the first known systematic review to examine the effectiveness of digital interventions in increasing the rate of flu vaccination amongst pregnant women. This review has the potential to inform whether digital interventions are an appropriate and successful method of increasing flu vaccination amongst pregnant women, and to determine which mode of digital intervention is most effective.

**Trial registration:**

This systematic review is registered on the international prospective register of systematic reviews (PROSPERO). Registration number pending.

## Background

Pregnant women and their unborn babies are at increased risk of complications as a result of flu, with pregnant women being at increased chance of hospitalisation and death due to physiological and immunological changes that happen during pregnancy [1–3], and unborn babies being at increased risk of premature birth, stillbirth and below average birthweight as a result of maternal flu [1]. Despite the increased risk of severe consequences from flu, and despite the flu vaccination having a good record of safety and effectiveness [4–6], uptake of the flu vaccination in England is annually below national targets, with 45.2% of pregnant women receiving the flu vaccination in the 2018/2019 flu season [7].

Access to internet use has increased rapidly over recent years, with 87% of all adults using the internet daily or almost daily during 2019, and with almost two thirds of households in the UK having access to mobile broadband [8]. The popularity of internet use is expected to continue to increase, with nearly 54 million people estimated to use an internet enabled smartphone in the UK by 2022 [9].

Digital interventions are one approach to increasing health behaviours that often utilise internet capabilities. Digital interventions have proven effectiveness in increasing behaviours such as smoking cessation [10], physical activity [11] and asthma self-management [12]. If digital interventions are effective in increasing flu vaccination amongst pregnant women, it suggests a mechanism for primary care services to impact the health of pregnant women and unborn babies, and reduce associated costs resulting from maternal flu. For the purposes of this review, the term digital behavioural interventions is defined as an intervention that attempts to change pregnant women’s vaccination behaviour that are delivered via a digital or mobile device directly to participants. This includes text messages (including text, video or audio based messages), interventions delivered by the internet (including by websites, mobile applications (apps) or social media sites), or other digital strategies [13].

To date, the effectiveness of digital interventions at increasing the rate of flu vaccination amongst pregnant women have yet to be determined. This review aims to establish whether digital interventions are effective at increasing flu vaccination amongst pregnant women, and to determine the size of the effect.

### Research question

How effective are digital interventions in increasing flu vaccination amongst pregnant women?

### Objectives

1. To examine the effectiveness of digital interventions in increasing flu vaccination amongst pregnant women.
2. To compare the effectiveness of different types of digital interventions in increasing flu vaccination amongst pregnant women

## Methods

Completed PRISMA-P checklist, showing recommended items to include in a systematic review protocol, and where in the protocol each item is located, can be found in Additional file 1.

### Eligibility criteria

Studies will be included that test the effectiveness of digital behavioural interventions in increasing the rate of flu vaccination amongst pregnant women. Comparators in included studies will be either usual care, a wait-list comparator, a historical control group where the digital intervention is not present, a digital intervention that is not about flu vaccination, or comparison to a non-digital intervention. The outcome being studied is the rate of flu vaccination amongst pregnant women. Only original research studies will be included, with any systematic reviews, protocols, commentaries and conference abstracts being excluded. Included studies will be either a randomised controlled trial, a non-randomised controlled trial, quasi-randomised controlled trial or other type of quantitative study that reports the rate of flu vaccination (for example, before and after trials) following the implementation of a digital intervention, which also contains a comparator group. Quantitative studies such as case series, case studies and case reports will also be excluded. No date or country restrictions will be placed on the search, but studies will be required to be published in English (Table 1).

**Table 1:** Inclusion and exclusion criteria

Characteristic	Inclusion criteria	Exclusion criteria
Population	Participants are pregnant women, over the age of 16 (or where there is a range of ages included, the majority of participants are over the age of 16).	Any participants other than pregnant women, or studies that include only adolescents (under the age of 16).
Intervention	Studies testing the effectiveness of a digital intervention (an intervention that attempts to change pregnant women’s vaccination behaviour that are delivered via a digital or mobile device directly to participants) to increase the rate of flu vaccination (if multiple types of intervention are tested, at least one of these needs to be a digital intervention, and results must allow for the rate of vaccination by a digital intervention to be extracted). Appropriate statistical information about the effectiveness must be provided	No intervention is tested, none of the tested interventions are digital
Comparator	Studies comparing the effectiveness of a digital intervention (for example, text message, website, mobile app) to usual care, to a wait-list comparator, to a non-digital intervention, to a digital intervention that is not about flu vaccination, or to a historical control group without digital intervention	No comparator, control, or usual care condition is present.
Outcome	Outcome being studied is the rate of flu vaccination (either actual vaccination behaviour or intention to vaccinate)	The rate of flu vaccination is not the outcome measure
Publication type	Original research studies only	Systematic reviews, protocols, commentaries, conference abstracts
Study design	Studies will be RCTs, non-RCTs, quasi-RCTs, or other quantitative study	Other study designs including quantitative studies that report audits, surveys and similar, and case series, case studies or case reports) or those that do not report the rate of flu vaccination after the implementation of a digital intervention.

### Outcome measures

The outcome measure will be the rate of flu vaccination amongst pregnant women after receiving a specific digital intervention, compared to the comparator group. This will either be self-reported flu vaccination status or rate obtained from electronic patient records.

In studies where the main outcome is intention to vaccinate rather than actual vaccination behaviour (uptake of vaccination), this rate will be used for this review.

### Information sources

Electronic bibliography databases will be searched during April and May 2020. MEDLINE, Embase, Web of Science, Scopus, Cochrane database, PsycINFO, and Cochrane Central Register of Controlled Trials (CENTRAL) will be searched for all eligible studies. Clinical trial registers will be searched for in-progress trials (e.g. ClinicalTrials.Gov) Search terms will include all possible terms relating to ‘vaccination’, ‘influenza’, ‘pregnancy’, and variations of ‘digital interventions’ to include interventions that contain significant influence from text messages, video, internet, or mobile phone applications (apps) [13, 14]. Boolean strategies of ‘AND’ and ‘OR’ will be employed.

Reference sections of included studies will also be screened by hand to identify any other eligible studies, and papers citing included studies will be searched.

### Search strategy

Specialist advice on the search strategy has been sought in developing the search strategy for this review, to ensure searches are comprehensive and capture all relevant studies in the searches. An example of the full search strategy for one database can be found in Additional file 2. Keywords will be searched in both titles and abstracts.

### Data management and screening process

Results of searches will be managed using Endnote software. Results from all databases will be combined, alphabetised and duplicates will be removed.

The first stage of screening will consist of all titles and abstracts being screened. Any studies that appear to meet the inclusion criteria will be subject to stage two of screening, which will consist of full text screening, where full papers will be obtained and screened against the eligibility criteria. Screening will be conducted by two researchers independently, and any discrepancies will be resolved with discussion. If a consensus is not reached, advice will be sought by a third researcher, until a full and final set of studies that meet the inclusion criteria are obtained. In addition to this, reference sections of all included studies will be screened for any studies that were not captured by the searches.

Data will be extracted from all included studies. This will be conducted by two researchers independently, using a pre-defined extraction form. Eligibility for inclusion in the meta-analysis will also be determined. The following information will be extracted from each included study: name of author, year of publication, study design, study setting, participants, details about intervention (including the mode of digital intervention such as text message, video, mobile phone app), comparison/control condition, rate of flu vaccination, and size of effect of intervention (if reported). Any discrepancies between data extraction carried out by the two researchers will be discussed, and a third author will be consulted if a consensus is not reached.

### Quality assessment

Risk of bias in Randomised Controlled Trials will be assessed using the Cochrane Risk of Bias tool [15]. Studies will be assessed on the domains below and classified as either low risk, medium risk, or high risk of bias:

1. Sequence generation
2. Allocation concealment
3. Blinding of participants, personnel and outcome assessors
4. Incomplete outcome data
5. Selective outcome reporting
6. Other sources of bias

Risk of bias in non-randomised controlled trials (including quasi-randomised controlled trials and quantitative studies) will be assessed using the Cochrane Risk of Bias in Non-randomised Studies of Interventions [16]. Studies will be assessed on the domains below and classified as either low risk, moderate risk, serious risk, critical risk of bias, or no information to make a judgement:

Pre-intervention

1. Bias due to confounding
2. Bias in selection of participants into the study

At intervention

3. Bias in classification of intervention

Post intervention

4. Bias due to deviation from intended intervention
5. Bias due to missing data
6. Bias in measurement of outcomes
7. Bias in selection of the reported result

### Data synthesis

Key information that is extracted from each included study will be synthesised. This will consist of descriptive information about the type and content of intervention in each condition as presented in the paper. The rate of flu vaccination will also be extracted from included studies and will be synthesised and discussed to determine if digital interventions are effective at increasing flu vaccination amongst pregnant women. Discussion about the risk of bias of included papers will also be included.

### Data analysis

Statistical information about the rate of flu vaccination in pregnant women will be presented, to establish whether digital interventions are more effective at increasing flu vaccination amongst pregnant women, than other types of interventions or comparison groups.

If studies report the rate of intention to vaccinate instead of actual vaccination behaviour (vaccination uptake), this data will be reported instead. Separate analysis for vaccination and intention to vaccinate will be conducted. If studies report both vaccination rate and intervention to vaccinate rate, then the actual vaccination rate will be used.

If sufficient statistical information is provided by included studies, a meta-analysis will be conducted to establish the pooled and weighted size of the effect of digital interventions in increasing flu vaccination amongst pregnant women. Standardised study effect sizes, using standardised mean differences (with 95% confidence intervals) will be calculated for the increase of flu vaccination rates in digital intervention conditions, compared to other intervention types or control/comparison conditions, using a random-effects meta-analysis model, where the statistical information provided allows. If applicable, separate meta-analyses will be conducted for vaccination intention and actual vaccination uptake. In studies where insufficient statistical information is include, study authors will be contacted to ask for further information. If no further information is provided, then the study will be excluded from the meta-analysis.

### Heterogeneity

As it is anticipated that interventions within included studies will differ, random effects model will be used for the meta-analysis. Heterogeneity between studies will be assessed using the chi-squared statistic and *I*^2^, to examine what percentage of variability between studies is due to heterogeneity rather than chance. Where *I*^2^ is over 40% moderate heterogeneity between studies is indicated [17].

### Subgroup analysis

If sufficient studies are included, and there is sufficient variation between the types of digital intervention used to try to increase flu vaccination amongst pregnant women, subgroup analysis will be performed to determine which type of digital intervention (for example, comparing text messages, mobile apps, websites) has the greater effect on increasing flu vaccination amongst pregnant women.

If there are sufficient studies, and sufficient variation between studies, additional sub-group analysis will be performed to determine the rate of self-reported flu vaccination verses validated flu vaccination (from medical records). This will be compared with the combined rate of flu vaccination, to determine if there is any difference between the rate depending on the way it is measured.

If there is sufficient variation in the risk of bias ratings of included studies, sensitivity analysis will be performed to determine whether results of the meta-analysis would change if high-risk studies were removed from the analysis.

### Publication bias

Funnel plots will be examined to identify the presence of publication bias. If missing studies as a result of publication bias are detected, a Trim and Fill analysis will be completed to account for the missing studies in the effect size [18].

## Discussion

Digital healthcare interventions are increasing in popularity and use, contributed by the increase in accessibility of technology and internet use. Examining the effectiveness of such interventions for flu vaccination amongst pregnant women provides useful insight into the research area.

### Limitations

The inclusion criteria set may restrict the number of studies that are eligible for the review. The review is examining only one type of vaccination (flu vaccination) in one population group (pregnant women), which will limit the number of papers, but will provide vital information about the potential role that digital interventions can play in increasing the uptake of flu vaccination for this vulnerable patient group.

Flu vaccination uptake is lower in pregnant women from low socio-demographic groups [19–21]. There is the possibility that a digital intervention is beyond the reach of some pregnant women within this lower socio-demographic group which may further increase the divide between pregnant women that do and do not have the flu vaccination.

### Strengths

To date, no review has examined the effectiveness of digital interventions in increasing flu vaccination uptake amongst pregnant women. A recent review has examined interventions that have been used to increase flu vaccination uptake, but current advancements in technology and internet use, and previous demonstrated effectiveness of digital health interventions [22, 10–12], it is relevant and timely to examine digital flu vaccination interventions.

### Potential implications

This review has the potential to increase current knowledge about whether digital interventions are likely to be an effective means of increasing flu vaccination amongst pregnant women and will hopefully highlight which modes of digital interventions are most effective. This knowledge will inform the design of future interventions, by allowing the most effective mode to be identified and used for maximum behaviour change.

### Registration of review

The protocol for this review was registered on the international prospective register of systematic reviews (PROSPERO). Registration number pending. Any amendments to the protocol will be amended on this registration.

## Supplementary information

**Additional file 1:.** PRISMA-P checklist

**Additional file 2:.** Example search strategy for Medline

## Data Availability

Not applicable
