# Supplementary material for: How effective are digital interventions in increasing flu vaccination amongst pregnant women? A systematic review protocol
Source: Syst Rev. 2020 May 28;9:117. doi: 10.1186/s13643-020-01372-z (PMC7254692; doi:10.1186/s13643-020-01372-z)
Supplement: Supplementary file 2 — Additional file 2:. Example search strategy for Medline [file 13643_2020_1372_MOESM2_ESM.docx]

Additional file 2: Example search strategy for Medline:

Database: Ovid MEDLINE(R) <1946 to March Week 4 2020>

Search Strategy:

--------------------------------------------------------------------------------

1 exp Influenza Vaccines/ or flu.mp. or exp Influenza, Human/ or influenza.mp. (104544)

2 exp Pregnancy/ or pregnancy.mp. (927256)

3 pregnant women.mp. or exp Pregnant Women/ (83211)

4 pregnan*.mp. (942979)

5 exp prenatal care/ or antenatal.mp. (50945)

6 exp maternal health/ or exp maternal welfare/ or maternal.mp. (277230)

7 2 or 3 or 4 or 5 or 6 (1043829)

8 1 and 7 (3634)

9 digital.mp. (105015)

10 apps.mp. (3394)

11 app.mp. (19703)

12 video*.mp. (140122)

13 telemedicine.mp. or exp Telemedicine/ (28921)

14 software.mp. or exp Software/ (242301)

15 computer*.mp. (758762)

16 exp Computers/ (77488)

17 web.mp. or exp Web Browser/ (86290)

18 telehealth.mp. (3411)

19 (text or texts or texting).mp. [mp=title, abstract, original title, name of substance word, subject heading word, floating sub-heading word, keyword heading word, organism supplementary concept word, protocol supplementary concept word, rare disease supplementary concept word, unique identifier, synonyms] (62785)

20 9 or 10 or 11 or 12 or 13 or 14 or 15 or 16 or 17 or 18 or 19 (1217156)

21 8 and 20 (91)
